# Development of Indirect Competitive Enzyme-Linked Immunosorbent Assay to Detect *Fusarium verticillioides* in Poultry Feed Samples

**DOI:** 10.3390/toxins11010048

**Published:** 2019-01-17

**Authors:** Aline Myuki Omori, Elisabete Yurie Sataque Ono, Melissa Tiemi Hirozawa, Igor Massahiro de Souza Suguiura, Elisa Yoko Hirooka, Maria Helena Pelegrinelli Fungaro, Mario Augusto Ono

**Affiliations:** 1 Department of Pathological Sciences, State University of Londrina, P.O. box 10.011, Londrina 86057-970, Paraná, Brazil; alineomori@gmail.com (A.M.O.); igormassahiro@hotmail.com (I.M.d.S.S.); 2 Department of Biochemistry and Biotechnology, State University of Londrina, P.O. box 10.011, Londrina 86057-970, Paraná, Brazil; eysono@hotmail.com (E.Y.S.O.); mel_hiro@hotmail.com (M.T.H.); 3 Department of Food Science and Technology, State University of Londrina, P.O. box 10.011, Londrina 86057-970, Paraná, Brazil; elisahirooka@hotmail.com; 4 Department of General Biology, State University of Londrina, P.O. box 10.011, Londrina 86057-970, Paraná, Brazil; mariafungaro@gmail.com

**Keywords:** Toxigenic fungi, polyclonal antibodies, immunoassay, mycotoxin, feed safety, food-borne fungi

## Abstract

Fumonisins are a group of toxic secondary metabolites that are produced by *Fusarium verticillioides* which are associated with poultry health hazard and great economic losses. The objective of the present study was to develop an immunological method to detect *F. verticillioides* in poultry feed samples. An indirect competitive enzyme-linked immunosorbent assay (ic-ELISA) based on a polyclonal antibody against 67 kDa protein of the *F. verticillioides* 97K exoantigen was developed to detect this fungus. Antibody anti-67 kDa protein showed cross-reactivity against *F. graminearum* (2–7%) and *F. sporotrichioides* (10%), but no or low cross-reactivity against *Aspergillus* sp. and *Penicillium* sp. exoantigens. The detection limit for the 67 kDa protein of *F. verticillioides* was 29 ng/mL. Eighty-one poultry feed samples were analyzed for *Fusarium* sp. count, 67 kDa protein of *F. verticillioides* and fumonisin concentrations. Eighty of the 81 feed samples (98.6%) showed *Fusarium* sp. contamination (mean 6.2 x 10^4^ CFU/g). Mean 67 kDa protein and fumonisin concentration in the poultry feed samples was 21.0 µg/g and 1.02 µg/g, respectively. The concentration of 67 kDa protein, as determined by ic-ELISA correlated positively (p < 0.05) with fumonisin levels (r = 0.76). These results suggest that this ic-ELISA has potential to detect *F. verticillioides* and predict fumonisin contamination in poultry feed samples.

## 1. Introduction

Brazil is the third largest corn producer in the world, and in the 2016/2017 harvest season, production reached 97.7 million tons [1]. Approximately 50% of corn production is intended for the animal feed industry (49 million tons) and 28.7 million tons are directed to the broiler feed industry [2].

Corn is the major ingredient of poultry feed, ranging from 55% to 58% of its composition. Because of its nutritional quality, corn is susceptible to contamination by toxigenic fungi, i.e., mycotoxin producers. 

Mycotoxins are secondary metabolites that are produced by filamentous fungi, which can cause acute and/or chronic toxic effects in both humans and animals at low concentration levels. *Fusarium verticillioides* (Sacc.) Nirenberg (synonym, *F. moniliforme* (J.) Sheldon); teleomorph, *Gibberella moniliformis* (synonym *G. fujikuroi* mating population A) is a primary corn pathogen and the main producer of fumonisins [3]. 

In addition to the serious economic losses for several commercial sectors, the natural occurrence of *Fusarium verticillioides* and fumonisins in corn and corn-based feed reduces the nutritional value of feedstuff and causes adverse effects on animal health and productivity. Fumonisins cause toxic effects on the liver, spleen, and kidney and are associated with immunosuppression, decreased weight gain, reduced mean egg production, and average egg weight in poultries [4,5].

Natural occurrence of *F. verticillioides* and fumonisins in corn and corn-based feed is a worldwide problem [6,7,8,9,10,11].

Traditional identification and detection methods for molds include culture in several media, microscopic examination, and chemical analysis of chitin, ergosterol, and secondary metabolites [12]. These methods show low specificity and sensitivity and they are time-consuming, except for the identification of secondary metabolites by chromatography and mass spectrometry. Although chromatographic methods show high sensitivity and specificity, they are laborious, use toxic and expensive reagents, and require an extensive clean-up process of the sample.

Several researchers have reported molecular methods, such as polymerase chain reaction (PCR) based on genes that are associated with the fumonisin biosynthetic pathway including *FUM1*, *FUM6*, *FUM8,* and *FUM13* [13,14,15,16,17]. Nevertheless, these methods were qualitative and group-specific detecting *F. verticillioides* in addition to other fumonisin or trichothecene-producing *Fusarium* species. On the other hand, Omori et al. [18] developed a PCR-ELISA based on the *FUM21* gene for *F. verticillioides* detection in corn, which was quantitative and showed sensitivity and specificity to *F. verticillioides* isolates.

An alternative method for fungi detection would be an Enzyme-Linked Immunosorbent Assay (ELISA), which allows for the analysis of several samples in a single test, shows simple sample processing and high sensitivity and specificity and it does not require toxic reagents. In addition, ELISA can detect the presence of fungi in food even after heat treatment, which enables the evaluation of contamination in processed foods. The ELISA, which uses immunogenic macromolecules produced and released to the culture medium throughout the growth of the fungus, known as exoantigens, is broadly employed for pathogenic fungi identification and detection once most fungi produce species-specific exoantigens [19]. 

Despite many efforts, the ELISAs developed to date to detect *Fusarium* species in food are genus-specific [20,21,22]. 

When considering that poultry feed contamination with *Fusarium verticillioides* is frequent, the serious economic losses, in addition to human and animal health hazard caused by mold contamination, it is essential to develop a method to detect fumonisin producing species to monitor the feed producing chain. Therefore, the objective of the present study was to develop an ELISA for *F. verticillioides* detection in the poultry feed.

## 2. Results and discussion

Within the *Fusarium* species complex (teleomorph *Gibberella fujikuroi*), which includes more than 50 species, *F. verticillioides*, *F. proliferatum,* and *F. subglutinans* are the main species that infect corn kernels [23]. *F. verticillioides* is the most common fumonisin producing *Fusarium* species infecting corn kernels [24,25,26,27,28]. In addition, some strains *of Aspergillus niger* have been reported as fumonisins B_2_, B_4_, and B_6_ producing species [29]. In the present study, an ELISA based on polyclonal antibody was developed to specifically detect *F. verticillioides* in corn-based poultry feed (matrix/substrate).

When considering the differences in geographic distribution and host/substrate preference of the various *Fusarium* species [30], in the present study the species were selected based on their occurrence in Brazilian corn, the major ingredient of poultry feed [24,26,28,31]. In Brazil, among 100 *Fusarium* isolates, a high frequency of *F. verticillioides* (96%) was reported in corn grains collected from four different regions, but *F. proliferatum* frequency (2%) was low [26]. Lanza et al. [28] performed morphological and molecular characterization of 230 *Fusarium* species that were isolated from corn grains from different geographic regions in Brazil and showed that *F. verticillioides* was the *Fusarium* species predominantly associated with corn grains (99%) in Brazil. Moreover, the occurrence of *F. proliferatum* (1%) was sporadic and *F. subglutinans* was not found in Brazilian corn. Therefore, in this study the species tested for cross reactivity was selected based on their occurrence (frequency) and host/substrate preference demonstrated in previous studies.

**Figure 1:**
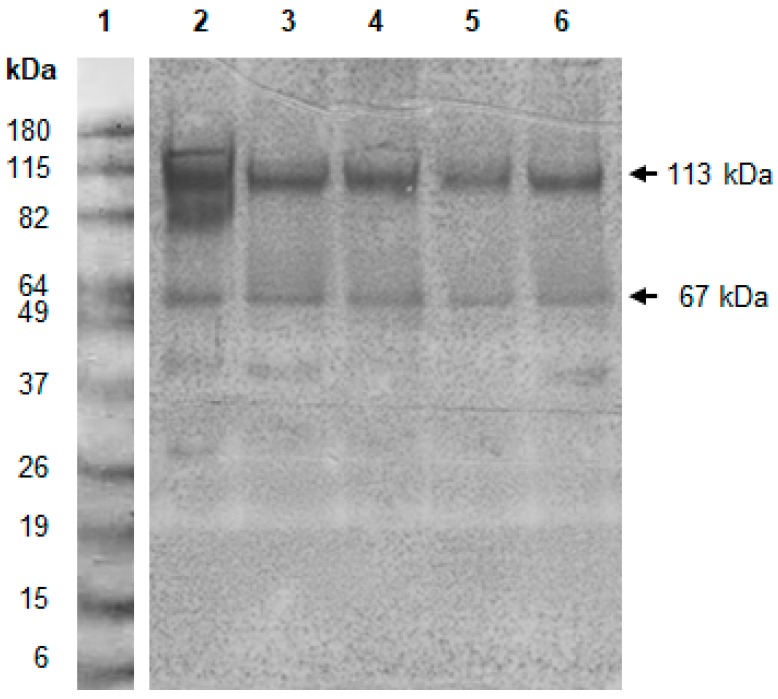
Reactivity of IgY Anti-97K exoantigen against the exoantigens of different *F. verticillioides* strains evaluated by Western blot: (**1**) Molecular weight standard, (**2**) 97K; (**3**) 119Br; (**4**) 104Ga; (**5**) 164G; and, (**6**) 103Br.

The chicken antibody (IgY) against the *F. verticillioides* 97K exoantigen was able to recognize different *F. verticillioides* proteins in Western blot (Figure 1), but showed cross-reactivity with the exoantigens of other fungal species, mainly *Fusarium* species, i. e., *F. sporotrichioides* (81%), *F. graminearum* (4–27%), *F. proliferatum* (26%), and *F*. *subglutinans* (17–19%) (Table 1). The cross-reactivity against exoantigens of *Penicillium* species ranged from 2% for *P. purpurogenum* exoantigen to 13% for *P. brevicompactum* exoantigen (Table 1). Moreover, the antibody showed 6% cross-reactivity only against *A. ochraceus* 153 strain and 3–10% against *A*. *carbonarius* exoantigens (Table 1). These results indicated the presence of similar epitopes in the *F. verticillioides* exoantigens and in the exoantigens of other fungal species. On the other hand, cross-reactivity was not observed against *A. niger*, *A. welwitschiae,* and *A. flavus* exoantigens (Table 1). Although there are several studies on ELISA for toxigenic *Aspergillus* sp. and *Penicillium* sp. in food [32,33,34], few reports on ELISA for *Fusarium* sp. [20,22,35,36] were found to discuss the current data. Biazon et al. [35] also produced polyclonal antibodies against *F. verticillioides* exoantigen, which showed cross reactivity with other *Fusarium* species, e.g., *F. graminearum* (51%), *F. sporotrichioides* (66%), and *F. subglutinans* (76%). Iyer and Cousin [20] developed an indirect ELISA based on polyclonal antibodies raised to the proteins that were extracted from the mycelia of *F. verticillioides*. These antibodies showed cross-reactivity against several *Fusarium* species, e.g., *F. graminearum* (67%), *F. sporotrichioides* (71%), and against *Monascus* sp. (43%) and *Phoma exigua* (51%). The authors concluded that the indirect ELISA developed would be promising for *Fusarium* sp. detection in grains or foods.

Several molecular methods have been reported, e.g., polymerase chain reaction (PCR) based on genes that are associated with the fumonisin biosynthetic pathway, including *FUM1*, *FUM6*, *FUM8*, and *FUM13* [13,14,15,16,17]. Nevertheless, these methods were qualitative and group-specific detecting *F. verticillioides* in addition to other fumonisin or trichothecene-producing *Fusarium* species. Dawidziuk et al. [14] developed a multiplex PCR to detect fumonisin (genes *FUM6*, *FUM8*), trichothecenes (genes *tri5, tri6*), and zearalenone (gene *zea2*)-producing *Fusarium* species. Toxigenic potential for fumonisins was detected with a sensitivity of 94% and specificity of 88% [14]. A multiplex PCR based on primers for the *FUM21*, *FUM1*, and *FUM8* genes [37] has been developed to distinguish toxigenic and non-toxigenic *Fusarium* sp. Divakara et al. [37] suggested that the *FUM21* gene showed better potential to distinguish fumonisin producer isolates from those non-producers. Omori et al. [18] developed a PCR-ELISA based on the *FUM21* gene for *F. verticillioides* detection in corn, which showed specificity to *F. verticillioides* isolates and a 2.5 pg detection limit.

The reactivity of the IgY antibody anti-*F. verticillioides* 97K exoantigen against the exoantigen of other *F. verticillioides* isolates, as evaluated by Western blot, showed the presence of two proteins with apparent molecular weights of 113 kDa and 67 kDa common to all of the isolates (Figure 1), suggesting that these proteins could be species-specific. Biazon et al. [35] also reported the presence of these two proteins in the exoantigens of *F. verticillioides* strains. In order to reduce cross-reactivity against the exoantigens of other fungal species, antibody against the 67 kDa protein was produced.

Antibody anti-67 kDa protein showed lower cross-reactivity to all the tested fungal species exoantigens as compared to the IgY antibody anti-*F. verticillioides* 97 exoantigen (Table 1). Cross-reactivity was not observed against *F. subglutinans*, *F. proliferatum*, *A*. *carbonarius*, *A. flavus*, *A. niger*, *A. ochraceus*, *A. welwitschiae*, *P. variabile*, *P. funiculosum,* and *P. brevicompactum* exoantigens and decreased from 81% to 10% for *F. sporotrichioides*, from 27% to 7% for *F. graminearum* 17102918 and from 2% to 1% for *P. purpurogenum* exoantigens when compared to the cross reactivity of IgY antibody against the crude exoantigen of *F. verticillioides* 97K (Table 1). These results suggest that the antibody against the 67 kDa protein of *F. verticillioides* 97K is more specific to *F. verticillioides*.

**Figure 2:**
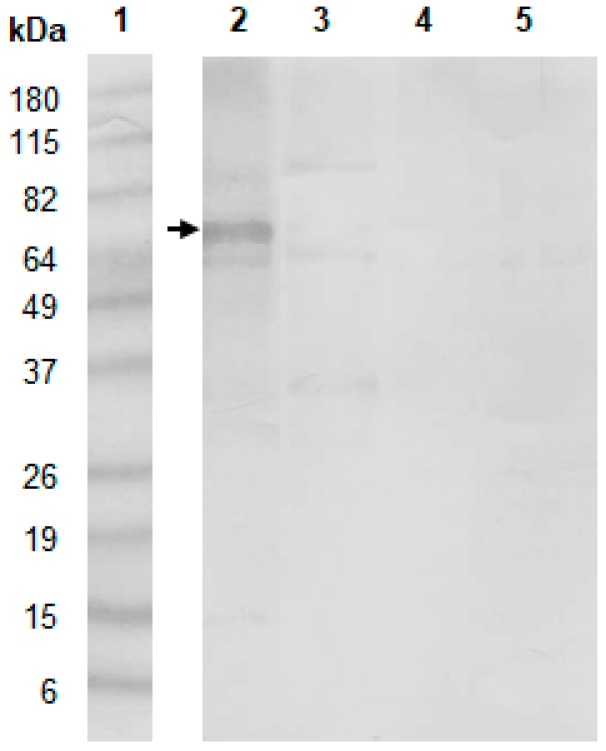
Reactivity of antibody anti-67 kDa protein against exoantigen of *F. verticillioides* 97K and exoantigens of other fungal species that showed cross-reactivity in the indirect ELISA evaluated by Western blot: (**1**) Molecular weight standard, (**2**) *F. verticillioides* 97K; (**3**) *F. sporotrichioides*; (**4**) *F. graminearum*; and, (**5**) *P. purpurogenum*. The arrow indicates the 67 kDa protein band.

The remaining cross-reactivity against exoantigens of *F. sporotrichioides*, *F. graminearum*, and *P. purpurogenum* with the antibody anti-67 kDa protein could be due to similar epitopes or the presence of the 67 kDa protein in these exoantigens. Western blot to evaluate the reactivity of the antibody anti-67 kDa protein against these exoantigens showed that the 67 kDa protein was not detected in these exoantigens, suggesting that the cross-reactivity probably occurred due to the similar epitopes that are present in other antigenic proteins (Figure 2). Although some cross-reactivity remained against *F. sporotrichioides* and *F. graminearum*, it would not be so problematic, when considering that the frequency of these fungal species in Brazilian corn and corn-based feed is very low. Moreover, *F. sporotrichioides* and *F. graminearum* are not fumonisin producers.

The ic-ELISA based on antibody against 67 kDa protein of the *F. verticillioides* 97K was able to detect and to quantify the antigen in poultry feed samples. The 67 kDa protein concentration in the samples ranged from 2.0 µg/g to 59.8 µg/g with mean of 21.0 µg/g (Table 2). Meirelles et al. [22] also developed an ic-ELISA and detected exoantigen ranging from 8.9 to 956.0 µg/g (mean of 217.3 µg/g) in corn samples. This difference in the concentration could be related to the type of antibodies used. In the present study, the antibody recognized the 67 kDa protein of the *F. verticillioides* exoantigen, whereas the antibody used by Meirelles et al. [22] recognized total *F. verticillioides* antigens and showed greater cross-reactivity against other species of *Fusarium*.

Fumonisins were detected in 89% (FB_1_) and 81.5% (FB_2_) of the poultry feed samples. FB_1_ levels ranged from 0.03 to 3.03 µg/g and FB_2_ levels from 0.03 to 1.27 µg/g (Table 2). Mean total fumonisin (FB_1_+ FB_2_) levels (Table 2) were higher than the pre-starter (0.77 µg/g) and grower (0.77 µg/g) poultry feed samples that were analyzed by Rossi et al. [8] but lower than those reported by Greco et al. [10]. Greco et al. [10] analyzed 49 poultry feed samples and detected fumonisins in 100% samples with mean levels of 1.75 µg/g. The maximum recommended fumonisin levels for laying hens and broiler feed are 30 µg/g and 100 µg/g, respectively [38]. Therefore, despite the high fumonisin frequency in feed samples, the levels detected in the present study are below the maximum limit that is allowed by the Food and Drug Administration [38].

Eighty of the 81 feed samples (98.6%) showed *Fusarium* sp. contamination with counts ranging from 50 to 7.5 × 10^5^ CFU/g (mean 6.2 × 10^4^ CFU/g) and 86% of the samples showed a contamination level ≤ 10^4^ CFU/g (Table 2). The mean *Fusarium* sp. counts (Table 2) was higher than those reported by Rossi et al. [8], who analyzed 158 pelleted feed samples, including four feed types and reported 1.3 × 10^2^ to 2.8 × 10^3^ CFU/g mean *Fusarium* sp. count. Labuda et al. [39] analyzed 50 samples of poultry feed mixtures of Slovakian origin and detected *Fusarium* sp. in 40% samples with 1.0 × 10^2^ to 1.0 × 10^5^ CFU/g count.

The 67 kDa protein concentration showed a weak positive correlation with the *Fusarium* sp. count (*r* = 0.24, *p* < 0.05) in the feed samples (data not shown). Yong and Cousin [33] reported similar results in a sandwich ELISA to detect aflatoxin producing *Aspergillus* species in corn. This may have occurred because the mold count is not a precise method for total biomass estimation, since only viable propagules can be detected and the degree of spore production affects the results [40].

**Figure 3:**
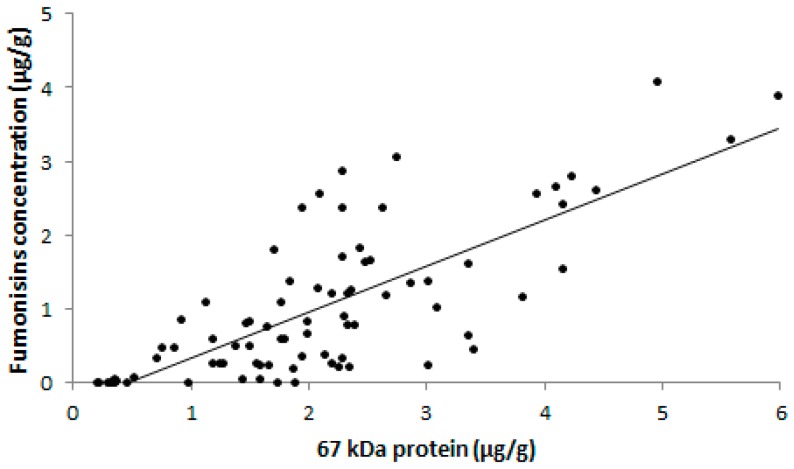
Correlation between fumonisin and 67 kDa protein concentration in poultry feed samples (*n* = 81) with the Pearson correlation coefficient of 0.76.

On the other hand, a good and significant positive correlation between the 67 kDa protein and fumonisin concentration was observed (*r* = 0.76, *p* < 0.05) in the feed samples (Figure 3). This suggested that the antibody anti-67 kDa protein detects mainly fumonisin producing *Fusarium* sp. and the ic-ELISA that is based on this antibody might be used to predict fumonisin contamination in poultry feed samples. 

Meirelles et al. [22] analyzed freshly harvested corn samples by ic-ELISA based on the antibody anti-*F. verticillioides* 97K exoantigen and observed weak correlation between exoantigen and fumonisin concentration, suggesting that the antibody recognized both the exoantigen of fumonisin producing and fumonisin non-producing *Fusarium* strains. The use of an antibody that is more specific to *F. verticillioides* probably contributed to the high positive correlation that was obtained in the present study.

In summary, the ic-ELISA based on an antibody specific to the 67 kDa protein is a promising method to detect *F. verticillioides* and predict fumonisin contamination in poultry feed samples.

## 3. Material and Methods

### 3.1. Fungal Isolates 

*F. verticillioides* isolates (97K, 119Br, 104Ga, 164G, and 103Br), *F. sporotrichioides*, *Penicillium variabile* 30 F 33-4, *P. funiculosum* 30 F 88-2, *P. purpurogenum* 30 F45, *Aspergillus niger* 10A, and *A. ochraceus* 153 belong to the Mycological Culture Collection of the Department of Food Science and Technology at the State University of Londrina. *F. graminearum* isolates (FSP27 and FRS26) were provided by the Mycological Culture Collection of Laboratory of Toxigenic Fungi and Mycotoxins of the Department of Microbiology of Biomedical Sciences Institute, University of São Paulo (São Paulo-Brazil); isolates of *F. graminearum* 17102918, *F. proliferatum* 559, *F. subglutinans* (332, 852), *A. flavus* (58A, 89A), *Aspergillus niger* (4138, 104CF, 23115), *A. carbonarius* (178, 180, 222), *A. ochraceus* (4363, 4368), and *A. welwitschiae* (112581, 115625) were provided by the Mycological Culture Collection of Department of General Biology, State University of Londrina, Paraná, Brazil. *Penicillium brevicompactum* was provided by the Institute of Food Technology (ITAL, Campinas, São Paulo, Brazil). All of the isolates were cultured in potato dextrose agar (PDA) at 25 °C. In the present study, the species were selected based on their occurrence in Brazilian corn, the main ingredient of poultry feed.

Production of fumonisins (FB_1_ + FB_2_) by *F. verticillioides* isolates was as follows: 97K (4050 µg/g), 103Br (1480 µg/g), 104Ga (3140 µg/g), 119BR (4050 µg/g), and 164G (3.59 µg/g). 

### 3.2. Exoantigen Preparation 

Exoantigens were prepared according to Biazon et al. [35]. Briefly, fungal spore suspensions were prepared in sterile deionized water with 0.1% Tween 80. The spore suspensions (10^7^ spores/mL) were inoculated in 1 L shaking flasks containing 250 mL brain heart infusion broth (BHI) and incubated at 28 °C and 150 rpm, for 14 days (*Fusarium* species) and seven days (other fungal species). The cultures were added with 0.02% thimerosal, incubated for 24 h at 4 °C, filtered, and centrifuged at 4500 × *g* for 20 min at 4 °C. The supernatants (exoantigens) were freeze-dried and stored at −20 °C.

The freeze-dried exoantigens were reconstituted in phosphate-buffered saline (PBS) and dialyzed first against deionized water and then against PBS for 24 h at 4 °C in dialysis tubing (12 to 16 kDa molecular cut off) and stored at −20 °C.

The exoantigen protein concentration was estimated using BSA as standard [41].

### 3.3. Antibodies Production 

Three laying hens (*Gallus gallus domesticus*) were inoculated with *F. verticillioides* 97K exoantigen homogenized with Freund’s incomplete adjuvant. Each animal was inoculated with three doses by intramuscular via with one-week interval between doses and the humoral response was evaluated by indirect ELISA.

The laying hen that showed the highest antibody titer against the *F. verticillioides* 97K exoantigen received a fourth dose five weeks after the third dose. The egg yolk reactivity of this animal was tested by indirect ELISA against the *F. verticillioides* 97K exoantigen and the IgY of the egg yolks with the highest reactivity was extracted with ammonium sulfate [42]. The IgY was analyzed by indirect ELISA and Western blot, and the protein concentration was determined using BSA as standard [43].

A rabbit (*Oryctolagus cuniculus*) was inoculated with slices of polyacrylamide gel containing the 67 kDa protein of *F. verticillioides* 97K [44]. The gel band was macerated in phosphate buffered saline (PBS) and mixed with Freund’s incomplete adjuvant. The animal was inoculated with three doses by via subcutaneous with one-week interval between doses and the humoral response was evaluated by indirect ELISA. The antibody anti-67 kDa protein was purified by affinity chromatography in HiTrap Protein G HP column (GE Healthcare, Piscataway, NJ, USA). A total of 18 mg of purified antibody anti-67 kDa was obtained from each 1.0 mL of immune rabbit serum.

This study was approved by Ethics Committee on Animal Experiments of State University of Londrina (CEEA/UEL, date of approve: 16 December 2009).

### 3.4. Indirect ELISA 

Indirect ELISA was performed to evaluate the reactivity of the rabbit serum, chicken serum, and IgY against 67 kDa protein or the *F. verticillioides* 97K exoantigen and to evaluate the cross-reactivity of the rabbit serum and IgY against the exoantigens of different fungal species (as described in 3.1. Fungal isolates). In brief, polystyrene microplates were coated overnight at 4 °C with 67 kDa protein (250 ng/well) or exoantigens (1.25 µg/well) in 0.1 mol/L carbonate bicarbonate buffer pH 9.6. After washing with 0.05% Tween 20 in PBS (PBS-T), the wells were blocked with 5% skim milk in PBS for 1 h. After washing with PBS-T, the rabbit serum, chicken serum, or IgY in 1% skim milk in PBS was added, and incubated for 1 h at 25 °C. The wells were washed with PBS-T, and anti-rabbit IgG-peroxidase conjugate or anti-chicken IgY-peroxidase conjugate was added, followed by incubation for 1 h at 25 °C. After washing with PBS-T, substrate-chromogen solution (H_2_O_2_/tetramethylbenzidine-TMBZ) was added. The reaction was stopped with 1 N H_2_SO_4_ and the absorbance was measured at 450 nm. All of the experiments were carried out in duplicate and the cross-reactivity was calculated, as follows:

(1) $$Cross-reactivity\;\left(\%\right)=\frac{A-B}{C-D}\times 100$$

where A is the absorbance of immune serum or IgY against exoantigen of the test fungus, B is the absorbance of pre-immune serum or egg yolk against exoantigen of the test fungus, C is the absorbance of immune serum or IgY against exoantigen of *F. verticillioides* 97K, and D is the absorbance of pre-immune serum or egg yolk against the exoantigen of *F. verticillioides* 97K.

### 3.5. Western Blot 

The reactivity of IgY anti-*F. verticillioides* 97K against the exoantigen of different strains of *F. verticillioides* (97K, 119Br, 104Ga, 164G, and 103Br) and the reactivity of the antibody anti-67 kDa protein against exoantigens of other fungal species were evaluated by Western blot. Briefly, the exoantigens were electrophoretically separated and transferred to a 0.45 µm nitrocellulose membrane. The membranes were then incubated for 1 h in 5% skim milk PBS with slight shaking. After washing with PBS-T, the membrane was incubated with IgY or antibody anti-67 kDa protein under agitation for 1 h. The membrane was washed with PBS-T and incubated with anti-chicken IgY-peroxidase conjugate or anti-rabbit IgG-peroxidase conjugate with shaking for 1 h. The membrane was washed with PBS-T and treated with substrate/chromogen solution (H_2_O_2_/diaminobenzidine) until bands revelation. The reaction was terminated by washing with distilled water.

### 3.6. Purification of 67 kDa Orotein by Affinity Chromatography

The 67 kDa protein was purified from the *F. verticillioides* 97K exoantigen by affinity chromatography using Cyanogen bromide-activated-Sepharose 4B (Sigma, Steinheim, NRW, Germany) resin linked to antibody anti-67 kDa protein purified. 

### 3.7. Poultry Feed Samples 

A total of 81 poultry feed samples (60% corn), from forty 500 kg batches, were collected from the experimental farm of the State University of Londrina, Northern Paraná State, Brazil. Sampling was performed at the beginning, middle, and end of each batch. Each sampling was carried out by collecting subsamples at many points at different depths in the box containing the feed. The samples were homogenized and then sent to the laboratory, ground to 50 mesh, and maintained at −20 °C until use.

### 3.8. Fusarium sp. Count and Exoantigen Extraction from Feed Samples 

Ten g of each ground feed samples were mixed with 90 mL sterile PBS and serial dilutions were carried out until 10^−4^ dilution. One mL of each dilution was added to Petri plates containing 25 mL PDA with chloramphenicol and tartaric acid and incubated at 28 °C for seven days. Subsequently, the genera were identified according to Nelson et al. [45] and *Fusarium* sp. was counted.

The remaining feed suspension was filtered (Whatman N° 1,Whatman GmbH, Dassel, NI, Germany) and stored at −20 °C for further quantification of *F. verticillioides* exoantigen by ic-ELISA.

### 3.9. Indirect Competitive ELISA 

The 67 kDa protein of *F. verticillioides* was detected and quantified in the feed samples by ic-ELISA, according to Meirelles et al. [22], with some modifications. In brief, the polystyrene microplate were coated with the *F. verticillioides* 97K exoantigen (0.4 µg/well) in 0.1 mol/L carbonate bicarbonate buffer pH 9.6 overnight at 4 °C. The wells were then washed and blocked with 1% skim milk PBS at 25 °C for 3 h. After washing with PBS-T, the microplate was incubated with antibody anti-67 kDa protein (225 ng/well) and feed sample extracts at 4 °C for 16 h. The microplate was washed with PBS-T and incubated with anti-rabbit IgG-peroxidase conjugate at 25 °C for 1.5 h. After washing with PBS-T the substrate solution (H_2_O_2_/TMBZ) was added. The reaction was stopped by adding 1 N H_2_SO_4_ and the absorbance was determined at 450 nm. All of the assays were carried out in duplicate and the results were expressed as the percentage of binding:

(2) $$Binding\left(\%\right)=\frac{Mean\;absorbance\;in\;the\;presence\;of\;soluble\;exoantigens}{Mean\;absorbance\;in\;the\;absence\;of\;soluble\;exoantigens}\times 100$$

The detection limit (LOD), quantification limit (LOQ), linearity, accuracy, and precision were calculated according to Rossi et al. [46]. The LOD and LOQ were determined, respectively, as three-fold and five-fold the standard deviation of absorbances from three replicate wells without 67 kDa protein. The linearity was determined by linear regression of three calibration curves (*R*^2^ = 0.98). The accuracy and precision were determined with ground feed samples spiked with 67 kDa protein at three concentrations (10, 20, and 30 µg/g). The values for LOD and LOQ were 29 and 33 ng/mL, respectively, and the linear range was 33 to 3125 ng/mL. The accuracy ranged from 85.3 to 99% (mean = 93%) and precision ranged from 4.2 to 10.3% (mean = 6.3%).

### 3.10. Fumonisin Analysis

Fumonisins B_1_ and B_2_ in poultry feed samples were determined by HPLC, according to Bordini et al. [11].

Sub-samples (10 g) of poultry feed were mixed with 30 mL methanol: water (3:1, *v*/*v*), shaken at 150 rpm for 30 min, and filtered (Whatman N° 1). One mL of the filtrate was applied to a Sep-Pak Accell Plus QMA cartridge preconditioned with 5 mL of methanol followed by 5 mL of methanol: water (3:1). After washing the cartridge with 6 mL of methanol: water (3:1) followed by 3 mL of methanol, the fumonisins were eluted with 10 mL of 0.5% acetic acid in methanol. The eluate was evaporated to dryness at 40 °C.

The sample residue was dissolved in 800 µL of methanol: water (3:1) and an aliquot (200 µL) were dried under nitrogen stream. After derivatization with 200 µL O-phthaldialdehyde reagent, HPLC injections were made within 1 min. Fumonisins were analyzed by a reversed-phase isocratic HPLC system (Shimadzu LC-10 AD pump and RF-10A XL fluorescence detector, Kyoto, KY, Japan), using a Luna C-18 Phenomenex column (250 × 4.6 mm, 5 µm, Scharlau, Barcelona, Spain). Excitation and emission wavelengths were 335 and 450 nm, respectively. The eluent was CH_3_OH: 0.1 mol/L NaH_2_PO_4_ (80:20, *v*/*v*) adjusted to pH 3.3 with orthophosphoric acid. The flow rate was 1 mL/min. The detection limit (LOD) and quantification limit (LOQ) were calculated as the minimum amount of toxin that could generate a chromatographic peak three and five times over the height/noise rate of the baseline, respectively. The LOD for FB_1_ and FB_2_ were 27.5 and 35.3 ng/g, and the LOQ for FB_1_ and FB_2_ were 45.8 and 58.8 ng/g, respectively. Recoveries of FB_1_ and FB_2_ from spiked feed samples in the range 100–1000 ng/g for FB_1_ and 150–800 ng/g for FB_2_ averaged 103.4% and 92.6% (mean CV 12.4% and 12.7%) and 108.0% and 94.6% (mean CV 16.8% and 18.8%), respectively, based on triplicate analyses. 

### 3.11. Statistical Analysis 

The correlation between 67 kDa protein concentration and *Fusarium* sp. count or fumonisin levels was analyzed by the Pearson correlation test. The statistical analysis was performed by the Statistica software version 7.0 (Statsoft Inc., Tulsa, OK, USA, 2008).

## Figures and Tables

**Table 1:** Cross reactivity of the IgY antibody anti-*F. verticillioides* 97K exoantigen, and the antibody anti-67 kDa protein of the *F. verticillioides* 97K with exoantigens of other fungal species evaluated by indirect ELISA.

Fungal Exoantigen	Cross-Reactivity (%)	
	Anti-Exoantigen	Anti-67 kDa Protein
*F. subglutinans* 332	19	0
*F. subglutinans* 852	17	0
*F. proliferatum* 559	26	0
*F. sporotrichioides*	81	10
*F. graminearum* FRS26	4	2
*F. graminearum* FSP27	8	0
*F. graminearum* 17102918	27	7
*A. niger* 10A	0	0
*A. niger* 104CF	0	0
*A. niger* 4138	0	0
*A. niger* 23115	0	0
*A. ochraceus* 153	6	0
*A. ochraceus* 4363	0	0
*A. ochraceus* 4368	0	0
*A. flavus* 58A	0	0
*A. flavus* 89A	0	0
*A. carbonarius* 178	3	0
*A. carbonarius* 180	0	0
*A. carbonarius* 222	10	0
*A. welwitschiae* 112581	0	0
*A. welwitschiae* 115625	0	0
*P. purpurogenum* 30 F45	2	1
*P. variabile* 30 F 33-4	7	0
*P. funiculosum* 30 F 88-2	10	0
*P. brevicompactum*	13	0

**Table 2:** *Fusarium* sp. count, 67 kDa protein and fumonisin concentrations in 81 poultry feed samples.

Parameters	Range	Mean	Median
*Fusarium* sp. count (CFU/g)	50–7.5 × 10^5^	6.2 × 10^4^	6.0 × 10^3^
67 kDa protein concentration (µg/g)	2.0–59.8	21.0	19.8
Fumonisin concentration (µg/g)			
FB_1_	0.03–3.03	0.69	0.64
FB_2_	0.03–1.27	0.33	0.30
Total (FB_1_+ FB_2_)	0.03–4.07	1.02	0.83
